# Risk-Targeted Selection of Agricultural Holdings for Post-Epidemic Surveillance: Estimation of Efficiency Gains

**DOI:** 10.1371/journal.pone.0020064

**Published:** 2011-05-25

**Authors:** Ian G. Handel, Barend M. de C. Bronsvoort, John F. Forbes, Mark E. J. Woolhouse

**Affiliations:** 1 The Roslin Institute and Royal (Dick) School of Veterinary Studies, The University of Edinburgh, Roslin, Midlothian, United Kingdom; 2 Centre for Population Health Sciences, University of Edinburgh Medical School, Edinburgh, United Kingdom; 3 Centre for Infectious Diseases, Ashworth Laboratories, University of Edinburgh, Edinburgh, United Kingdom; Massey University, New Zealand

## Abstract

Current post-epidemic sero-surveillance uses random selection of animal holdings. A better strategy may be to estimate the benefits gained by sampling each farm and use this to target selection. In this study we estimate the probability of undiscovered infection for sheep farms in Devon after the 2001 foot-and-mouth disease outbreak using the combination of a previously published model of daily infection risk and a simple model of probability of discovery of infection during the outbreak. This allows comparison of the system sensitivity (ability to detect infection in the area) of arbitrary, random sampling compared to risk-targeted selection across a full range of sampling budgets. We show that it is possible to achieve 95% system sensitivity by sampling, on average, 945 farms with random sampling and 184 farms with risk-targeted sampling. We also examine the effect of ordering samples by risk to expedite return to a disease-free status. Risk ordering the sampling process results in detection of positive farms, if present, 15.6 days sooner than with randomly ordered sampling, assuming 50 farms are tested per day.

## Introduction

After the apparent end of an animal disease epidemic, a country will normally benefit from demonstrating that infection is no longer present in its livestock. This is both to satisfy international trade requirements [1], [2] and also to identify previously undiscovered infection to prevent recrudescence. Demonstration that infection has been controlled also has domestic, societal and political advantages. The process is described as *demonstration of disease freedom* [3] .With many diseases this process includes a prescribed sampling of livestock in the vicinity of the previously affected premises. Appropriate diagnostic tests are used to determine the serological status of sampled animals and hence infer the infection status of the whole region. Unless all animals are simultaneously sampled with a perfect diagnostic test there will be uncertainty in the subsequent estimate of a region's infection status. Much work has been done to determine optimal sample sizes and performance limits of such surveys [3], [4], [5], [6], [7], [8]. These studies assume random sampling of animal holdings, usually within a defined surveillance zone that would normally surround the previously infected premises. Current disease control and surveillance policies generally involve the definition of a surveillance zone as a buffer at a prescribed radius around previously detected, infected premises. In the case of foot-and-mouth disease in the United Kingdom this is a 10 Km radius zone around any designated infected premises. For the purposes of illustration and discussion this study will focus on the design of post-foot-and-mouth disease epidemic surveys although the approach may be generalised to other diseases.

Post-epidemic demonstration of freedom from foot-and-mouth disease is informed by guidelines from The World Organisation for Animal Health (OIE). Previously these guidelines have been prescriptive regarding the sampling strategy. Changes in OIE guidelines have increased flexibility allowing a more pragmatic approach to design, provided that the survey adequately supports the claim of disease freedom [1].

The original guidelines required farms to be selected from within the surveillance zone on a random basis to achieve an expected survey system performance (e.g. a 95% confidence of detecting an infected farm if infection is present at a predetermined design prevalence such as 2%). Within each selected farm, samples are taken to achieve a within-farm expected survey performance; typically to detect infection on the farm with a 95% probability if it were present at some previously defined, within-group, design prevalence (often 5%). In the case of disease control by vaccination, current procedures may advise testing of all vaccinated animals on all vaccinated farms [9], [10]. Diagnostic tests have imperfect specificity, including those optimised for vaccinated animals, and farms may occasionally be classified as previously infected when they are not. These false positive farms will normally be screened by further confirmatory diagnostic testing.

Our study examines the traditional approach for post epidemic surveillance for disease freedom using random selection of farms within the surveillance zone and suggests a more efficient methodology. This targets farm selection using a model of un-discovered infection risk to choose which farms to sample and when to sample them. This approach is expected to reduce sampling costs by requiring fewer farms to be tested and to expedite a region's return to disease-free status by finding infected farms, if they are present, more quickly. Risk based approaches have been previously discussed for identification of disease freedom from Trichinella in pigs [11] and Scrapie in sheep [12]. These studies showed significant benefits to risk-targeted sample selection. Our study considers the application of risk-targeted surveillance to a post-epidemic population.

## Methods

In this study we discuss the estimation of surveillance system performance from an estimated risk of undiscovered infection on each animal holding. Then we compare the performance of risk-targeted and random surveillance systems in a post-epidemic setting using available data.

### Surveillance system sensitivity

The purpose of post-epidemic sero-surveillance is to inform a decision about the disease status of a region. A requirement of classification of a region as free-of-disease is surveillance evidence of no previously infected animals or circulating infection. In this analysis we consider that the performance of a survey to demonstrate disease freedom is assessed by the probability that it detects infection or circulation of infection in a region if infection is present. This is the system sensitivity (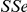) [13]. For this analysis we assume that an optimally efficient survey design either:

1. Maximises, for a given budget, the probability (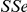) of detecting any infected but previously undetected (hereafter referred to as ‘undiscovered infected’) animals in the area, if present, or;
2. Minimises the sampling cost of a survey that achieves a desired probability (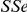) of detecting undiscovered infected animals, if present, in the area.

The system sensitivity 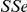 is defined:
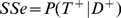
(1)Where 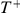 is the event of at least one farm testing positive in the region, 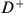 is the event of at least one farm being infected but not detected prior to the start of post-epidemic serological surveillance and 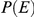 is probability of an event 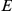.


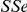
 can be estimated by assuming that the test system has perfect system specificity as discussed in [14]. That is, any initially test-positive farms are retested until they are demonstrated to be genuine positive or cannot be shown to be negative. Effectively the case definition for a test positive farm is a farm that tests positive after confirmatory and follow-up retesting, irrespective of its true infection status.

So generally with a diagnostic system:
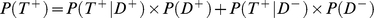
Assuming perfect system specificity:
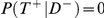
So:
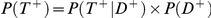
hence
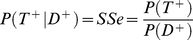
(2)To estimate the system sensitivity (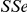) of different post epidemic sampling strategies using equation (2) it is necessary to estimate 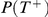:
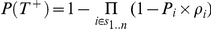
Where 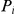 is the probability that farm 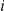 is infected and undiscovered, 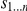 are the indices of a sampled set of 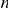 farms from the population of 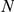 farms and 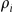 is the farm level sensitivity on farm 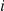 (i.e. the probability of a positive farm result if an infected farm is selected, sampled and tested). We assume that 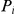 and 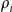 are independent, i.e. the sensitivity with which infection is detected on a farm is independent of the risk that the farm is infected and undiscovered.


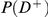
, the probability that undiscovered infected animals remain in the region is estimated:
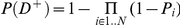
Giving:
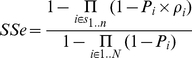
(3)


### Risk-targeted versus random farm selection

If all farms have an equal probability of being infected and undiscovered, the survey has a constant farm level sensitivity across all farms and the cost of sampling farms is constant there will be no advantage of risk-targeted sampling over random sampling. However if the probability of infection, 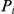, or farm sensitivity 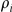 vary then maximum system sensitivity within a sampling or cost constraint will be obtained by selecting a sample set 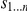 of 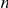 farms to maximize:
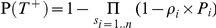
As 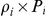 is always positive this is maximised by maximizing 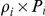. If the sampling constraint is simply a number of farms, 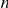, to be sampled this gives a sample set of the first 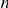 farms when they are ordered by decreasing 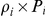.

If the sampling constraint is a fixed sampling cost budget and it is assumed that all farms have an equal sampling cost (cost of visiting the farm plus cost of sampling on the farm) then the most efficient sample will be the selection of as many farms as possible choosing farms in order of decreasing 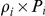 until the budget constraint is met.

The efficiency gains from risk-targeted sampling will depend on the form of the distribution of undiscovered infection risk. In the following section we adapt a previously published model for risk of infection and use it to estimate potential gains in surveillance system efficiency resulting from application of risk-based as opposed to random sampling in a post-epidemic surveillance scenario.

### Study scenario - Devon, UK 2001 foot-and-mouth disease epidemic

We estimated the performance gains of risk-targeted surveillance using historical data from the foot-and-mouth outbreak in Devon, UK in 2001. The post-epidemic surveillance analysis is confined to sheep only as this was the species subject to widespread sero-surveillance. To estimate the gains of risk-targeted surveillance we need an estimate of risk of undiscovered infection for the candidate farms. In this example the risk of infection is estimated using a statistical model [15].. Our analysis also requires an estimate of probability that infection escapes discovery during the outbreak period. In this example this was estimated from post-outbreak surveillance data. In future application of risk-targeted surveillance the discovery model results would have to be assumed to be exchangeable across similar outbreaks or estimated from local or small-scale surveillance data.

### Epidemic model

The foot-and-mouth disease outbreak in Devon 2001 was estimated to start on the 17th February and end with animals on the last known infected premises culled on 19th June 2001. One hundred and seventy two premises were declared as ‘infected premises’ based on clinical signs and subsequent confirmation during the outbreak. Disease was controlled with movement restrictions and by culling and disinfection of the infected premises and other designated high-risk holdings. Sero-surveillance during and after the epidemic identified a further 15 farms in this area as infected but otherwise undiscovered (these farms were recorded in the DEFRA submission to the OIE to substantiate freedom from disease and hence recorded in the disease control system database [16], [17]).

Diggle et al. [18] use a partial likelihood approach to parameterise a model of daily infection probability for each farm in Devon based on the modelling framework from Keeling et al. [19]. This model will hereafter be referred to as the ‘transmission model’. From the transmission model, the probability, 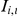, that an uninfected farm 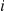 becomes infected on day 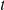 of the epidemic is estimated using formula 4.
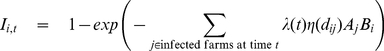
(4)Where:
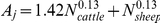


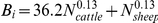


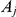
 represents the transmission potential of an infected farm 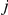, 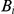 the susceptibility of farm 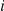. 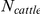 and 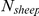 are the stocking numbers of cattle and sheep respectively. The parameter 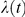 is a baseline hazard. The distance kernel 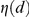, representing the decreasing risk of transmission between farms with increasing Euclidean separation, 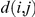 is modelled:
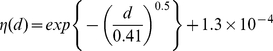
(5)Transmission model parameters were as used in [18] except the baseline hazard (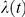), which represented the overall risk of transmission occurring in the Devon area, varying with time, during the epidemic. In Diggle (2006) [18] this was estimated from the daily case report data (approximately 5×10^−5^ with slight variation over the course of the epidemic). This baseline hazard would be dependent on stocking densities, husbandry practices, foot-and-mouth disease virus serotype, time of year and other factors. For the Devon region the baseline hazard was relatively constant with time so was kept fixed in our model for simulation purposes.

We used this modified transmission model to estimate the day-by-day probability of infection in each of the 4856 premises in Devon recorded as having sheep in the June 2000 agricultural censuses conducted in England and Wales by the Ministry of Agriculture, Fisheries and Food (Department for Environment Food and Rural Affairs (DEFRA) from June 2001. The simulation used the estimated infection and culling dates from the 172 premises designated as ‘infected premises’ during the outbreak in 2001 from the disease control system (DCS) database [17] as potential sources of infection.

There are two scenarios by which an infected farm will become a farm with undiscovered infection by failure to observe clinical disease: one is if infection on the farm is not clinically detectable i.e. disease is effectively sub-clinical; the other is if clinical signs are present but are not observed. The latter scenario is partially time dependent, there being a limited period during which infection may be clinically detected (of up to several weeks duration). As serological surveillance will normally start several weeks after the last clinically observed case and the frequency of cases in the tail end of the epidemic is low the time dependent effects may be disregarded and the two modes by which a farm may be missed considered as one probability. It is necessary to estimate a non-discovery multiplier to estimate the probability that an infected farm is not detected and becomes an undiscovered infected farm. This probability may be different for every farm and is likely to change over the course of the epidemic and post-epidemic period as surveillance efforts vary. Unfortunately only limited data are available to estimate this parameter for each farm.

### Estimation of probability of discovery of infection

The post epidemic sero-surveillance in Devon after the 2001 epidemic involved sampling and testing of some 4,407 farms from approximately 4,500 remaining sheep farms [16] using a well validated solid phase ELISA test with confirmatory virus neutralisation testing [20]. We assume that all farms that could be serologically discovered were discovered. Sero-surveillance identified ten holdings as sero-positive in Devon after the slaughter date of the last infected premises (we have not considered the five serologically detected premises prior to this date as representative of infections not discovered during the epidemic). One hundred and seventy two holdings were identified as infected premises during the epidemic in Devon having been detected either clinically or by in-epidemic sero-surveillance. Exploratory data analysis suggests that farms that were discovered during the epidemic were more likely to have cattle present and more likely to be close to other infected premises. We used a logistic regression approach, modelling the log odds of a farm being serologically discovered as a linear function (equation 5) of risk factors to estimate the discovery probability across farms and also to capture the large uncertainty in the associated parameter estimates resulting from estimation based on only 10 positive cases . The probability of discovery 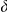 of an infected farm was modelled:
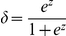


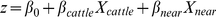
(5)Where 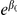 is the baseline odds of discovery, 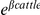 the odds ratio for discovery if cattle are present and 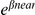 the odds ratio for discovery if the farm is within 3 Km of an IP. 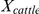 and 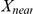 are indicator variables for presence of cattle and adjacency to an IP. This model is hereafter referred to as the ‘discovery model’.

Given the limited number of serologically discovered farms in the data set, binary predictor variables of cattle presence (i.e. cattle numbers greater than zero) and adjacency of less than 3 Km to the nearest infected premises (IP) were used to reduce the model complexity. The 3 Km adjacency cut-off was selected as regulations require there to be a heightened surveillance during an epidemic in the zone within 3 Km of infected premises and hence there is likely to be a higher probability that an infected farm is discovered. The probability of detection is assumed to remain fixed for each farm over the course of the epidemic.

The parameters of the discovery model were estimated from the sero-surveillance results for Devon in 2001 using a Bayesian approach with vague priors for the risk factor coefficients. The Bayesian posterior estimates were generated with Monte Carlo Markov chain simulation in JAGS software [21] called from the R Statistical system [22] retaining 5000 sets of samples from the posterior distribution of the parameters after discarding an initial set of 5000 simulations. Alternative parameterisation and the inclusion of different predictors were compared using the Deviance Information Criterion [23]. Chain convergence was assessed using the Brooks, Gelman and Rubin statistic [24].

Draws from these posterior distributions were then used with demographic data from the 2001 Agricultural census (as a sampling frame and source of stock/location data) to estimate the mean probability of discovery, if infected, for each sheep farm in Devon. Daily probability of infection from the transmission model and probability of discovery if infected from the discovery model are combined to estimate the daily probability for each sheep farm that the farm has become infected and that the infection has not been detected. This assumes that the probability of discovery and probability of infection are independent, conditional on the risk factors used in the transmission and discovery models. i.e.
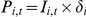
The resulting daily probability of farm 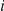 becoming infected yet not clinically detected is converted to a probability that farm 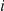 has become infected yet not clinically detected at any point during the epidemic, 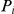:
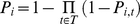
(6)Where 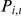 if the probability of undiscovered infection on farm 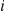 on day 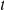, estimated with the transmission and discovery models and 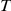 is the duration of the epidemic.

### System sensitivity versus number of farms sampled

The system sensitivity (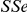) of risk targeted surveys is estimated using a range of farm sample sizes (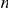) from just one farm to the whole population, by preferentially selecting the 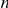 farms with the highest probability of undiscovered infection using equation 6. For this estimation the farm-level diagnostic sensitivity (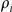) was set at 95%. For comparison the expected system sensitivity for randomly selected samples is estimated by simulation. The random sampling is assumed to take place from within the 3 Km protection and 10 Km surveillance zones. For each sample (size *n*) of farms from 1 to the remaining, post-epidemic, population of 3526 sheep farms, within the protection and surveillance zones, 1000 random samples of size *n* were drawn (without replacement). The system sensitivity of each sample was calculated using equation 3 and the mean for each sample size stored.

### Zonal location of sampled farms

Conventionally farms are sampled from within the surveillance and protection zones. This may not be the most efficient approach; it is possible that farms outside these zones may be at higher risk of undiscovered infection and hence should be sampled with priority. To investigate this farms were classified by decreasing probability of undiscovered infection according to their location within their zones. The zones used are the current 3 Km protection and 10 Km surveillance zones constructed using the respective buffers around the 172 infected premises from the Devon 2001 epidemic.

### Consequences of imperfect information

The above methodology makes the assumption that the overall model used to estimate each farm's probability of undiscovered infection is correct i.e. the transmission model is precise and unbiased and that the discovery model's estimate of probability of discovery is exchangeable between epidemic settings. It does not assume perfect information about each farm's undiscovered infection status but perfect information about each farm's probability of undiscovered infection. In reality, any model estimating the probability of infection in a farm will have error.

With risk-targeted sampling, it is the rank of estimated probabilities of undiscovered infection and their heterogeneity that determines the choice and expected benefits of farm selection. A model that estimates the probabilities as a monotonic increasing function of the true probabilities will still be able to perfectly inform selection of an optimal sampling set (though give an incorrect estimate of its system sensitivity) for a given sample size. Sub-optimal farm selection will only occur when the model causes incorrect selection of lower probability farms for inclusion in the sample. Imperfect models that correctly rank the farms' risk of undiscovered infection will, however, incorrectly estimate the system sensitivity achieved by a survey.

To investigate the effect of model uncertainty we explored two approaches to adding an error component to the overall estimate of probability of undiscovered infection derived from the transmission and discovery models. One approach modifies the estimated probability of infection for each farm by adding normally distributed error on the log-odds scale. The other randomises a proportion of the estimated probabilities so that only a proportion of the farms' have an accurately estimated risk of infection. The estimates with added error are then used to select farms and with the original, added error-free risk estimates, used to estimate system sensitivity performance of the resulting simulated datasets. These approaches are described in detail below.

### Transformed normal error approach

The transmission model and discovery model are used to estimate the probability of undiscovered infection 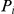 for each farm 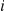. This value is then transformed to the log-odds scale and a normally distributed error component is added. The result is then re-transformed to the probability scale:
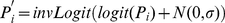
(7)Where 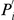 is the transformed probability of undiscovered infection on farm 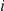 and logit and invLogit are the logistic and inverse logistic transformations respectively. For σ greater than zero this will add an error component to the model's predictions. For a range of σ from 0 to 25 the estimated probabilities with error added as in equation 7 were used to select farms for sampling and the probabilities from the original model (with no error component added) were used to estimate the resulting system sensitivity (performance). This estimation was repeated 1000 times to provide an estimate of the mean performance with each level of overall prediction error.

### An alternative partial knowledge approach

Rather than simulating errors in the estimation of each farm's risk, we also estimate the performance of risk-targeted sampling if the risk is only correctly known for a proportion of farms. This was done for proportions from 1 (i.e. perfect knowledge) to 0 (i.e. no information on risk of undiscovered infection).

### Effect of risk-targeted sampling on delay to declaration of disease freedom

Given a selected set of farms for sero-surveillance sampling (whether by the above transmission and discovery model-driven method or by another, e.g. random sampling or a veterinary expert directed method) there may be flexibility to choose the order with which farms are visited and sampled. Sampling and subsequent sample handling, analysis and recording may introduce delays of several days between sampling and result. If a sampled farm tests positive for infection and virus is subsequently isolated on a farm it may be classified as a new outbreak and consequently disease freedom may not be declared until a fixed period has elapsed after the culling of stock on this farm. Hence it is desirable to order sampling such that farms that are most likely to have undiscovered infection will be visited, sampled and analysed first.

To estimate the potential benefits of ordered sampling we estimate time from start of sampling to identification (on average) of the last positive farm for sero-surveillance in Devon. A sample set of farms of a given size is selected, either at random from the whole population, or by decreasing risk of undiscovered infection using the transmission and discovery models. Sampling from these farms is then simulated over a surveillance period assuming that 50 farms can be sampled each day. In the risk-targeted approach high-risk farms are sampled first. In the random approach the farms are sampled in a random order. Over repeated simulations the status of sampled farms is simulated (using a bernoulli process with probability equal to the farm's probability of undiscovered infection). For all farms this status is combined with their simulated sample timing data to give the time that each positive farm was sampled. Repeated over the simulation set the maximum of this time delay gives an estimate of the time from start of sampling to the sampling of the last positive farm for each approach.
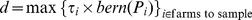
(8)Where 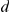 is the date of sampling of last positive farm, 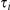 the sampling date for farm 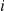 and 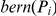 a Bernoulli random variable with probability 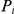. We assume that 50 farms are sampled and tested each day. The simulation was repeated 5000 times for a range of sample sizes and the mean of the latest dates stored.

The transmission and discovery models, subsequent estimations of risk and evaluations of system sensitivity were calculated with the R Statistical System [22].

## Results

### Results from discovery model

Farms with cattle present were more likely to be detected, if infected, during the epidemic, as were farms within 3 Km of a previously infected farm. The magnitude of these estimates has a large uncertainty. Detailed results are shown in Table 1.

**Table 1:** Results from Bayesian discovery model - mean and SD of coefficients of logistic model (and odds ratio) predicting discovery of an infected farm.

Variable	Mean	SD	Odds
Cattle present^1^	3.11	0.97	22.5
Near IP (≤3 Km)^2^	2.67	0.87	14.4

1 Are any cattle recorded as present on the holding according to the agricultural census?

2 Is the holding in question within 3 Km of a previously determined infected premises?

### Overall estimated of risk of undiscovered infection

**Figure 1:**
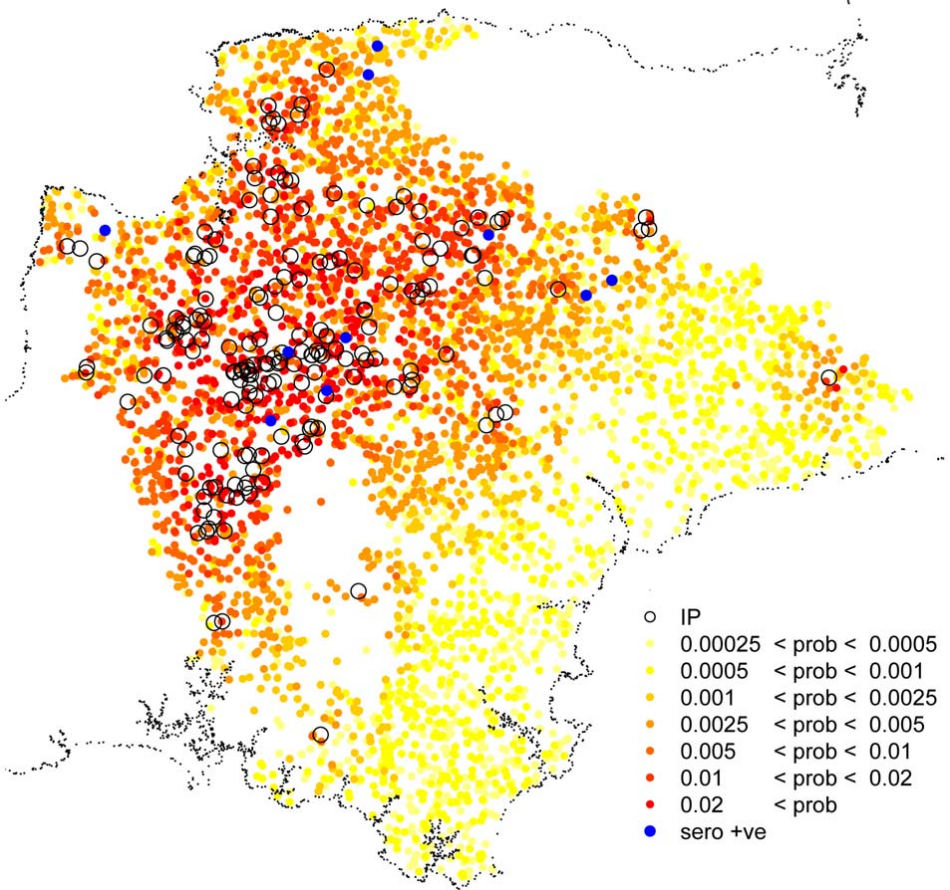
Risk-map for undiscovered infection. Estimated risk that individual sheep/mixed farms may be infected but undiscovered with foot-and-mouth disease at the end of the UK 2001 outbreak in the county of Devon. Farms classified as infected premises (IP) in 2001 are shown as black circles. The ten farms found to be sero-positive in 2001 after the epidemic are also shown (blue dots).

The estimates of post-epidemic undiscovered infection combine the transmission model with a farm-specific estimate of discovery of infection from the discovery model. The model estimated an expected 11.2 infected but not discovered farms in Devon with 7.8 within the protection zone (0–3 Km from the nearest IP), 3.0 within the surveillance zone (3–10 Km from the nearest IP) zones and an expected 0.4 farms outside these zones. The results are summarised, by zone, in Table 2. The spatial distribution of risk of undiscovered infection is shown in Figure 1. Farms with a high risk of undiscovered infection are spatially associated with the premises that were identified as infected during the epidemic. The spatial component of risk of undiscovered infection is a combination of the increased risk of farms near to detected infected premises and the increased probability of undiscovered farms being further from detected infected premises. The results suggest that the modelled infection risk overwhelms the estimated discovery risk such that, overall, farms near to detected infected farms are more likely to be infected but undiscovered.

**Table 2:** The mean result of 500 simulations for the 3 km protection zone, the 10 km surveillance zone and the whole region.

Zone	Total farms^1^	Cumulative farms	Expected undiscovered farms^2^	SSe (all farms in zone)^3^
Protection zone (0–3 Km)	1439	1439	7.79	0.976
Surveillance zone (3–10 Km)	2087	3526	3.01	0.821
Other (>10 Km)	1330	4856	0.381	0.337

Results are given for each zone and cumulatively across zones. They are the number of farms, the expected number of undiscovered infected farms and the system sensitivity if all farms in the zone were sampled with an on-farm survey of 100% sensitivity.

1 The total number of animal holdings within the zone.

2 The expected number of farms in the zone that will have an undiscovered infection.

3 The estimated system sensitivity to detect previous infection in the entire region if all the farms in the particular zone are sampled and tested.

### Performance of risk-targeted sampling

**Figure 2:**
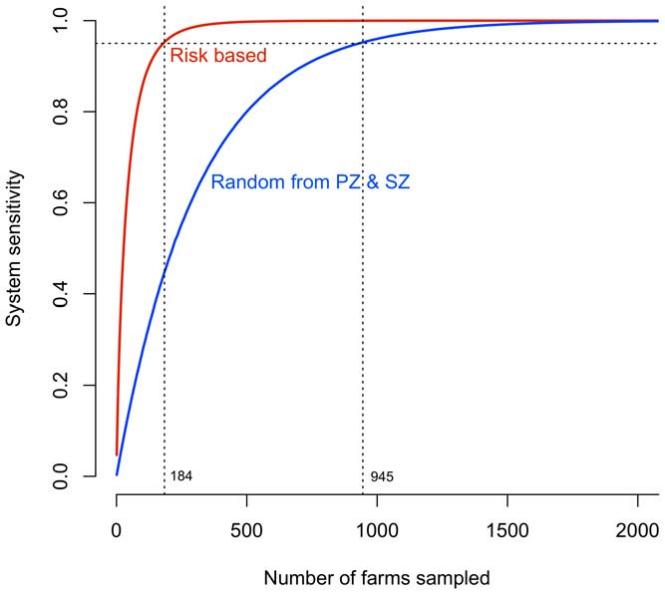
Performance of random and risk-targeted sampling. Comparison of system sensitivity (SSe) of random sampling from the protection (PZ) and surveillance zones (SZ), a 10 Km buffer around designated infected premises (blue line) and risk-targeted sampling (red line). Horizontal dashed line is at 95% system sensitivity (SSe), the conventional target for region level post-epidemic surveillance. The vertical lines dotted lines show corresponding sample sizes required to achieve 95% SSe for the two approaches.

The comparative results of risk-targeted sampling and random sampling are shown in Figure 2. Risk-targeted sampling gives a markedly better performance than random sampling for the same sample size (number of farms visited); for example, assuming the combination of transmission and discovery models provides perfect information about a farm's probability of undiscovered infection, only 184 farms need to be sampled to give a 95% system sensitivity as compared to 945 farms if random sampling is used. These system sensitivity performance gains relative to sample size would either increase the probability that the survey system detects disease, if present, or reduce the cost of surveillance to attain a required (95%) system performance. The relative gains of employing risk-targeted sampling decrease as the sample size increases until approximately 2000 farms are sampled when both risk-targeted and random sampling converge onto a system sensitivity of 1. The improvement in performance using risk-targeted sampling was found to be robust to error in the risk estimate; risk-targeted sampling was still more efficient than random sampling from the protection and surveillance zones when the risk status was known for only 20% of the farms.

### Zonal sampling

**Figure 3:**
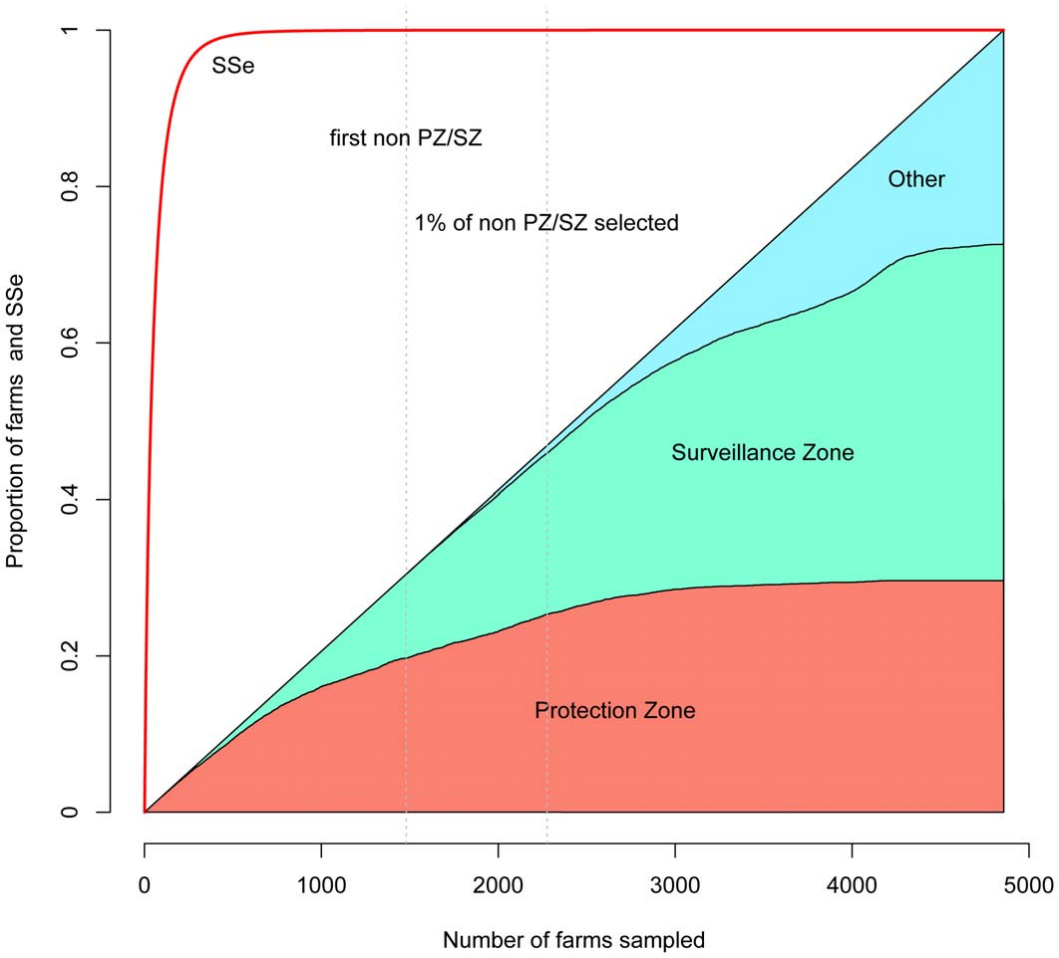
Risk-targeted sampling and traditional surveillance zones. Source zone of farms when the farms are allocated in decreasing probability of undiscovered infection. Vertical dotted lines showing points at which first and first 1% of farms outside the surveillance zone (SZ – a 3–10 Km buffer around designated infected premises) and the protection zone (PZ – a 3 Km buffer around designated infected premises) will be selected. The red line shows the estimated system sensitivity (SSe) using risk-targeted selection for the range of sample sizes.

Conventionally, sampling has drawn samples, unless veterinary judgement suggests otherwise, from within the 10 Km surveillance zone. Figure 3 shows the zone that samples would be taken from when risk-targeted sampling is used with the potential to draw from any farm in the restricted area. As sample size increases to 1476 farms the first farm outside the protection and surveillance zones will be selected. By a sample size of 2254 only 1% of farms will be selected from the area outside the surveillance zone. These sample sizes represent system sensitivities of virtually 100% and as such would be unlikely to be required for regulatory purposes. These results suggest that the conventional approach of sampling within the surveillance zone is rational if random selection within a geographical buffer zone is a regulatory requirement.

### Effect of ordering on delays to disease freedom

**Figure 4:**
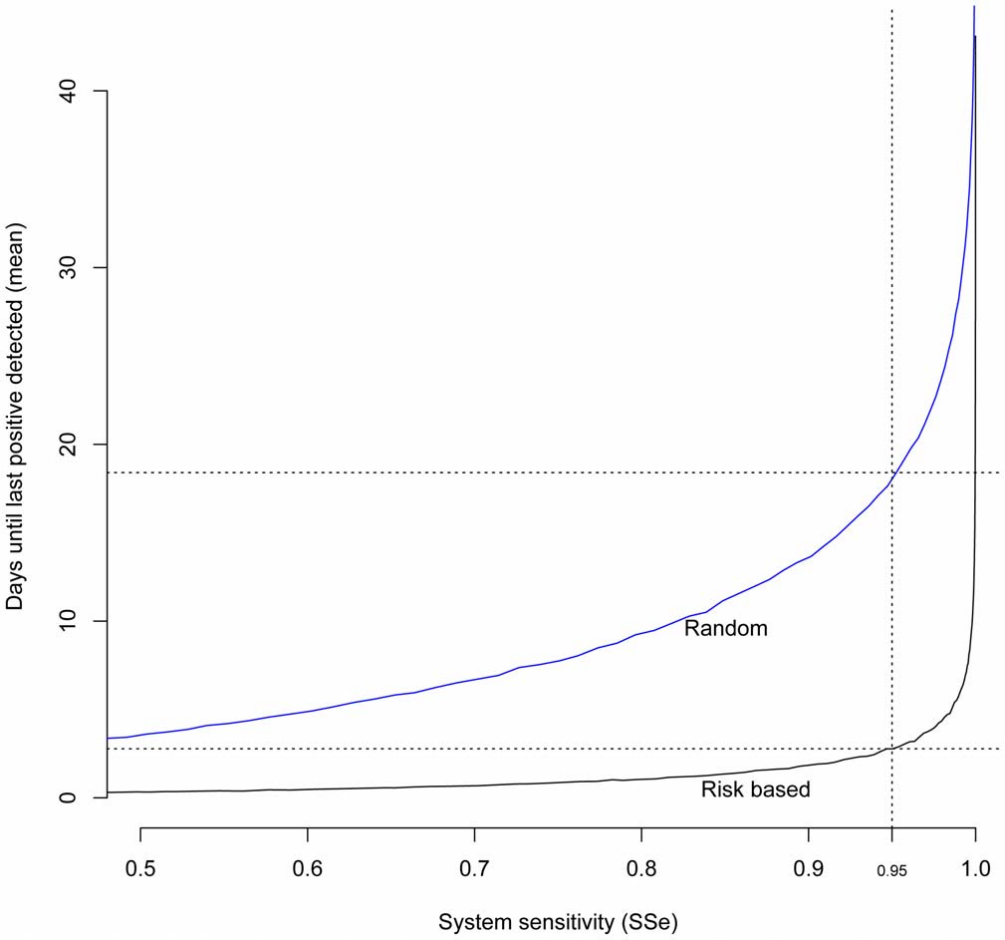
Timing benefits of risk-targeted and ordered sampling. Expected time to last positive farm for randomly selected/ordered (blue) and risk-targeted and ordered sampling (black) shown plotted against system sensitivity (SSe). This analysis assumes that 50 farms are sampled and tested per day.

The time to last positive sample is plotted against the system sensitivity for a risk-ordered and randomly ordered approach to farm sampling in Figure 4. The result demonstrates that risk-targeted sampling and ordering, given a desired system sensitivity, will result in any farms that turn out to be test positive being selected and sampled markedly sooner than if ordering is not used. For a target system sensitivity of 95%, risk-targeted selection and ordering (assuming 50 farms can be sampled and tested per day) will, on average, mean that the last positive farm is sampled 2.8 days after sampling commences whereas random selection will mean that the last positive farm is sampled 18.4 days after sampling starts. Detection of a test positive farm will result in follow up confirmatory tests and possible disease control consequences such as further investigation of contact farms and animal culling. Hence risk-targeted ordering may decrease delays to ultimate declaration of disease freedom in a region that has experienced a disease outbreak. Furthermore previously undiscovered infected farms may be viable sources of onwards transmission so prioritising detection of such farms with application of appropriate disease control may reduce the probability of disease reappearance compared to random ordering.

## Discussion

This study shows that a suitable model of infection risk can be used to target and order sero-surveillance sampling to increase efficiency and reduce delays to declaring disease freedom after a foot-and-mouth disease outbreak. Historically, post-epidemic surveillance for foot-and-mouth disease has targeted sampling to farms within the 3 Km protection zone and 10 Km surveillance zone, presumably on the basis that any undetected infected farms are most likely to be in these areas. This involves an implicit spatial assumption about disease transmission that may be derived from veterinary assessment. Risk-targeted selection refines this to target specific farms within the vicinity of the outbreak/epidemic. The benefits of this technique are shown to be potentially large with an 80% reduction in sample sizes for the same system performance (95% SSe) and an expected 85% reduction in time to last positive case.

### Model uncertainty

The realization of these potential benefits is dependent on the accuracy with which a predictive risk model can order farms by undiscovered infection risk. We have used simple approaches to model uncertainty to explore the consequences of imperfect knowledge of farm infection risk. This shows that the benefits of targeted sampling were reasonably robust to the introduction of noise/uncertainty into model estimates; risk-targeted sampling with knowledge of only 20% of farms' infection risk will still result in a better performance than surveillance-zone targeted sampling. As long as a risk model has some information value it will increase efficiency and reduce delays to declaring disease freedom.

However, if a predictive model of farm infection risk is particularly poor (i.e. almost random in its predictions) its application could result in a survey with efficiency and timing worse than a randomly sampled and ordered survey that draws from the traditional (overall high risk) surveillance and protection zones. Whilst such a scenario is unlikely it means that the risk-targeted sampling and ordering approach should be used cautiously, especially with models that are potentially over-fitted, unstable or otherwise suspect.

### Assumption of 95% farm sensitivity

For the comparisons of performance we have assumed that all farms' livestock are sampled and tested to give a farm level diagnostic sensitivity of 95% in accordance with previous OIE codes. Depending on the size of the farm and the available diagnostic tests the cost of reaching this performance will vary from farm to farm and may even be unobtainable when the individual animal diagnostic test sensitivity is lower than the farm level performance target and the farm size is small [25]. If this constraint were removed then a survey design would have freedom to choose not only which farms in the region to sample but rather which animals in the region to sample and indeed which diagnostic test to use and how to interpret the results for each sample. Although the removal of these constraints would give a potentially lower cost method of achieving a required regional performance it is a high dimension optimisation problem that is computationally difficult. Furthermore, the resulting varying farm-by-farm performance and sampling requirements may be politically harder to justify to both farmers and decision makers.

### Discovery probability model

To estimate the probability that a farm is infected but has not been detected we have used a model that estimated probability of infection and detection and a simple model of discovery probability. The discovery model used a distance greater than 3 Km to an infected farm and presence of cattle as farm risk factors. The calculation of probability of undiscovered infection using the simple product of this non-discovery probability and the infection risk assumes that these two probabilities are independent, *conditional*, on the risk factors included in both models. It is possible, though, that other predictors such as location, farm size, farm area, other stocking factors and husbandry factors will have a joint influence on infection risk and discovery probability. These factors may increase or decrease the variation in undiscovered infection risk and hence mean that the estimated benefits of risk-targeted selection are incorrect. Whilst it is impossible to estimate this effect without more data, it is unlikely given the large numerical range of infection risk probabilities that the conditional effects would be unlikely to significantly alter the order of estimated benefits and hence the risk-targeted selection method is still likely to be beneficial in comparison to random sampling.

We used a logistic regression framework for the discovery model. In some scenarios such as an epidemic that is controlled early with possibility of large numbers of undiscovered infection other approaches may be valuable such as the inclusion of non-discovery within the transmission modelling framework as explored by Chis Ster et al. [26].

### Probability of historical infection and risk of onwards transmission

This analysis has used the classical approach to the declaration of disease freedom where by all evidence of infection or circulation of infection is treated equally. Recent sero-conversion is more likely to be associated with infection that may result in onwards transmission. Weighting for this has not been included in the analysis but could be incorporated into risk targeted designs by using a time increasing weighting factor on risk and hence prioritising selection of potentially more recently infected farms.

### Concluding remarks

Current, random sample based surveillance is likely to be inefficient requiring more farms to be visited and more animals to be sampled than necessary to achieve a given performance target. Risk-targeted selection of farms for post epidemic surveillance is more efficient and will also expedite the process of declaration of disease freedom. Prompt detection of undiscovered infected farms may also reduce the risk of onwards transmission and recrudescence of the outbreak. The technique of risk-targeted surveillance may be applied to different post epidemic setting, such as after FMD controlled by vaccination or for the elimination of other diseases such as Blue Tongue Virus however such applications would require evaluation and application with different models of transmission and discovery probability. Whilst risk-targeted selection is robust, to give the best and most reliable gains, risk models also need to be robust and precise hence continued research will be required to model the disease and surveillance processes to best inform such surveillance strategies.
